# Emerging Evidence for the Importance of Dietary Protein Source on Glucoregulatory Markers and Type 2 Diabetes: Different Effects of Dairy, Meat, Fish, Egg, and Plant Protein Foods

**DOI:** 10.3390/nu8080446

**Published:** 2016-07-23

**Authors:** Kevin B. Comerford, Gonca Pasin

**Affiliations:** California Dairy Research Foundation (CDRF), 501 G Street, Ste. 203, Davis, CA 95616, USA; pasin@cdrf.org

**Keywords:** dairy, milk, dairy protein, whey protein, plant protein, animal protein, type 2 diabetes mellitus, blood glucose, insulin, bioactive peptides

## Abstract

Observational studies provide evidence that a higher intake of protein from plant-based foods and certain animal-based foods is associated with a lower risk for type 2 diabetes (T2DM). However, there are few distinguishable differences between the glucoregulatory qualities of the proteins in plant-based foods, and it is likely their numerous non-protein components (e.g., fibers and phytochemicals) that drive the relationship with T2DM risk reduction. Conversely, the glucoregulatory qualities of the proteins in animal-based foods are extremely divergent, with a higher intake of certain animal-based protein foods showing negative effects, and others showing neutral or positive effects on T2DM risk. Among the various types of animal-based protein foods, a higher intake of dairy products (such as milk, yogurt, cheese and whey protein) consistently shows a beneficial relationship with glucose regulation and/or T2DM risk reduction. Intervention studies provide evidence that dairy proteins have more potent effects on insulin and incretin secretion compared to other commonly consumed animal proteins. In addition to their protein components, such as insulinogenic amino acids and bioactive peptides, dairy products also contain a food matrix rich in calcium, magnesium, potassium, trans-palmitoleic fatty acids, and low-glycemic index sugars—all of which have been shown to have beneficial effects on aspects of glucose control, insulin secretion, insulin sensitivity and/or T2DM risk. Furthermore, fermentation and fortification of dairy products with probiotics and vitamin D may improve a dairy product’s glucoregulatory effects.

## 1. Introduction

Experts may still argue the science and semantics of whether a “calorie is a calorie” when it comes to effects on body weight; however, it is abundantly clear that all calorie sources are not created equal when it comes to their effects on metabolic parameters such as glycemia, insulinemia, fat oxidation, and protein synthesis. At a basic level, dietary carbohydrates, fats and proteins all provide energy which can be measured in heat units such as calories, but more importantly, the different macronutrients come in many different forms which can lead to very different fates and functions in the body. The digestive, absorptive and metabolic fate of a macronutrient depends on a combination of factors which include the macronutrient’s particular chemical structure, the food matrix they are part of, the other foods and medications they are consumed with, and the nutrigenetic and nutrigenomic profile of the consumer. Additionally, the ways in which the body responds to macronutrients can change drastically depending on its nutrient and energy status, activity levels, stress response and disease states.

Individuals with type 2 diabetes mellitus (T2DM) generally lose their ability to secrete insulin in response to carbohydrates; however, they are able to retain, or potentially even increase their ability to secrete insulin in response to protein and amino acid ingestion [1,2]. For example, protein intake has been shown to be equally as effective as carbohydrate intake at stimulating insulin secretion in subjects with T2DM [3], whereas in healthy subjects it has been shown to only be one-third as effective as glucose at stimulating insulin secretion [4]. However, not all protein sources are created equal in their abilities to modulate insulin secretion and insulin sensitivity [5]. Moreover, there are many more factors involved in glycemic management than just insulin. In addition to the glucoregulatory pancreatic hormones amylin and glucagon, the incretin hormones—glucagon-like-peptide-1 (GLP-1) and glucose-dependent insulinotropic polypeptide (GIP)—play a role in potentiating the insulinotropic actions of pancreatic islet beta cells [6]. Compared to healthy subjects, individuals with T2DM appear to retain normal GLP-1 functions, but have a lesser ability of GIP to influence insulin secretion [7]. However, certain dietary proteins, peptides and amino acids can potently stimulate both GIP and GLP-1 secretion in healthy and T2DM populations [1,3,8,9,10]. Additionally, the ingestion of different sources and forms of protein with—or before—a meal can have diverse effects on postprandial insulin and glucose concentrations in subjects with T2DM [11,12,13,14]. Furthermore, blood glucose is regulated by more than postprandial changes in endocrine hormones. Recent evidence suggests that the amino acid leucine may also influence glucose sensing pathways in the hypothalamus, thereby regulating whole-body glucose and energy metabolism in ways that are not currently well understood [15,16]. Non-insulin-dependent glucose regulation by specific amino acids could prove to be an important mechanism for glucose control in insulin-resistant individuals such as those with T2DM. Taken together, these findings suggest that there are several dietary protein qualities that can modulate glucoregulatory pathways in subjects with T2DM. The objective of this paper is to review the current scientific evidence regarding the effects of different animal and plant food protein sources on T2DM risk and glucoregulatory markers in human subjects.

## 2. Protein Classifications and Quality Scores Are of Limited Use for Optimizing Protein Intake for Blood Glucose Regulation

To date, the most commonly used categorizations of dietary proteins are according to their essential amino acid (EAA) content (i.e., complete vs. incomplete), their basic taxonomy (i.e., plant-based vs. animal-based), or their particular function in a plant or animal (i.e., signaling vs. structural). Of these grouping systems, the broad taxonomic distinction of plant-based and animal-based protein has been the most commonly studied. Nuts, seeds, legumes, and certain ancient grains (e.g., quinoa, kamut, amaranth, etc.) are some of the plant foods highest in protein content. Soybeans in particular, are much higher in protein and EAAs than other plant-foods [17]. However, with the exception of soy, most plant proteins are limited in one or more of the amino acids lysine, threonine, tryptophan, cysteine or methionine. Combining different plant proteins has been shown to be an effective way of attaining the necessary ratio of amino acids for meeting human requirements [18]. Most animal protein sources, with the exception of collagen/gelatin, contain adequate proportions of all the EAAs necessary to meet human requirements. Each protein source (e.g., soy, milk, meat, egg, etc.) is composed of various protein fractions with different properties, and many of those fractions can be further divided into groups of individual proteins that differ in their composition, structure and function (Table 1 and Table 2).

The source of a protein is often used as a surrogate for its quality (i.e., animal sources are generally high quality, while plant sources are generally low quality). Traditionally, the quality of proteins has been ranked by assessing their biological value, nitrogen balance dynamics, protein efficiency ratio and/or limiting amino acids (Table 2), but there are also other quality factors that should be addressed when assessing protein quality in the context of optimal health and metabolic disease management. These include a protein’s complete amino acid profile, non-protein nutritional profile, bioactive properties, amino acid absorption rate, insulinogenic properties, and overall effects on glycemia [30]. For example, the total amount of EAAs in a protein is often times the only consideration in assessing its quality, but the ratio of EAAs in a protein drastically alters its effects on metabolism. The EAA leucine, which is also classified as a branched chain amino acid (BCAA) due to its chemical structure, is more insulinogenic than other EAAs. Additionally, leucine is the only EAA which has been shown to affect glucose sensing in the brain [15,16], as well as stimulate muscle metabolism by both insulin-dependent and insulin-independent mechanisms [31]. In short, current protein classification systems and protein quality measures are poorly suited for optimizing protein intake in the context of preventing or managing metabolic disease [32].

While the individual nutrients in foods, such as protein or fats, can have profound effects on health, simply studying individual nutrients in isolation does not account for the total effects of a food. Many foods also have bioactive effects beyond their nutrients, which can influence health. For example, some nutrients and bioactive compounds in the diet can interact within foods, and between foods, in ways that are currently not well understood [33]. The idea of protein optimization should therefore be considered in the context of the whole food consumed, along with its potential additive, synergistic and inhibitory interactions with other foods in the diet.

The current body of evidence comparing plant and animal protein intake on glycemic control and T2DM risk has produced inconsistent results. A major reason for the inconsistencies is that protein foods can be compared in several different ways, either matched for weight, energy content, protein content, or by normally consumed portion size. Each type of comparison will potentially provide different results. Furthermore, there are many different types of commonly consumed plant proteins (e.g., soy, nut, seeds, beans, peas, lentils, etc.) and animal proteins (e.g., meat, milk, fish, eggs, etc.) that have been studied, each with their own set of protein quality characteristics and non-protein components. For example, when compared to animal-based proteins, most plant-based proteins have higher concentrations of non-protein compounds that are associated with improved health outcomes, such as fiber, magnesium, potassium, and antioxidant phytonutrients [30]. Therefore, plant sources of protein may have several beneficial effects associated with the prevention and management of T2DM, but these benefits may be independent of the actual type or amount of protein in them. Overall, the protective effects of plant protein foods on T2DM risk appear to be indirect, most likely provided by their ability to improve related risk factors such as body weight, blood pressure, lipids, and inflammatory markers, which in turn can attenuate insulin resistance [34,35,36]. In agreement with this concept, the 2010 Dietary Guidelines Advisory Committee (DGAC) reported that “the limited data collectively suggests that vegetable protein does not offer special protection against type 2 diabetes” [37]. In their more recent report, the 2015 DGAC did not revisit this particular topic [38].

While there are few known differences in glucoregulatory qualities between the various types of plant-based proteins [30], there are several important qualitative distinctions between animal-based proteins (Table 1 and Table 2). These distinctions are based on the type of animal (e.g., cow, chicken, fish, etc.) producing the protein, and on the types of proteins produced by the animal (e.g., meat, milk, egg). For example, the muscle proteins produced by a cow and fish share similar structures and functions, but they have evolved to adapt to different environments and will therefore differ in protein, peptide, and amino acid composition [39,40] (Table 1). Furthermore, the protein content and composition within different muscles of the same animal will also vary (e.g., light meat compared to dark meat in a chicken), as will the different proteins within any particular muscle (e.g., myofibrillar proteins are considered high quality complete proteins, while the stromal proteins in muscle are considered a low quality incomplete proteins [41] (Table 1). Eggs also contain structural proteins, along with hundreds of other functional proteins that have evolved to assist with various physiological processes revolving around embryonic nourishment and development [42]. The specific protein fractions are very different between the egg yolk and white [20] (Table 1). Milk proteins differ considerably from structural proteins and embryonic development proteins in both their functions and composition. Milk proteins are highly specialized for immune protection, cell signaling and nutrient transfer—containing higher levels of associated minerals, BCAAs, insulinogenic amino acids, and bioactive properties than other animal proteins [43,44].

## 3. Different Effects of Plant-Based and Animal-Based Protein Sources on Glucoregulatory Markers and Type 2 Diabetes Risk

Prospective results from a pooled analysis of adults from the Nurses’ Health Study (*n* = 72,992 women), Nurses’ Health Study II (*n* = 92,088 women), and Health Professionals Follow-up Study (*n* = 40,722 men) showed that higher plant protein intake was associated with a moderately decreased risk of T2DM. Results also showed that replacing 5% of energy intake from animal protein foods with plant protein foods reduced the risk for developing T2DM by approximately 20%–25% [45]. When the five animal protein foods (dairy, poultry, eggs, red meat, processed meat, and fish) were assessed individually in comparison to plant protein intake, dairy was the only animal protein food group on par with plant protein. The other five animal protein foods were associated with higher risk for T2DM, compared to plant protein foods. Replacement of one serving of plant protein with one serving of poultry, eggs, fish, or red meat resulted in a 9%–13% increase in T2DM risk, while replacement with processed meat resulted in a 21% increased risk. Similarly, the European Prospective Investigation into Cancer and Nutrition (EPIC) InterAct case-cohort study, which included data on eight European countries, found that T2DM risk varied with the total amount and specific type of protein consumed [46]. The results from this study showed no effect of plant protein intake on T2DM incidence, while a 10 g higher daily intake of total protein was associated with a 13% increased incidence of T2DM, and a 10 g higher daily intake of animal protein was associated with a 12% increase in T2DM incidence. The EPIC InterAct researchers did not find any differences between the types of animal proteins consumed (e.g., meat, dairy, fish) and T2DM risk. However, when the relationship of total meat, dairy, and fish intake and T2DM in this population was studied by a different set of researchers, higher intakes of red and processed meat were found to be associated with an 8% and 12% increased risk for T2DM, respectively [47], while overall fish and dairy intake were not [48,49]. Further analysis showed that intake of specific types of fish (i.e., fatty fish) and dairy products (i.e., cheese and fermented dairy products) were inversely associated with T2DM risk, suggesting that in addition to protein content, there are several other nutritional and/or bioactive factors that may moderate the relationship between diet and T2DM risk.

A recent systematic review and meta-analysis of 13 randomized controlled trials investigating the effects of replacing animal protein with plant protein on glycemic control in subjects with diabetes found that a ratio favoring higher plant protein intake resulted in better glycemic control [50]. Animal proteins and plant proteins varied across the randomized controlled trials (RCTs), limiting the analysis between dietary protein sources and types. Dietary protein type analysis showed a large reduction in fasting glucose when total animal proteins were replaced with plant proteins, and a much smaller reduction when dairy or meat proteins were replaced with plant proteins. The researchers theorized that lower heme-iron intake and/or higher l-arginine intake in the higher plant protein consumers may explain some of the differences in glucose regulation between the groups. A major confounder in this meta-analysis was that the majority of studies included high levels of soy protein products, including soy protein supplements, which are not commonly consumed in American populations. Soybeans contain >35% protein, and soy protein supplements contain up to 95% protein, while most other plant-based proteins range from 2% to 25% protein [17]. With the exception of soy-based foods and supplements, there has been limited research on the intake of high-protein plant foods or plant protein supplements in subjects with T2DM [51].

Intervention studies comparing the glucoregulatory effects of plant, fish, and dairy proteins on healthy subjects have been limited to very specific protein sources (e.g., soy protein, lean fish, and cottage cheese) and may not be representative of other protein sources in these groups. A randomized isocaloric challenge study comparing cottage cheese to soy protein isolate and lean cod fish protein was conducted on 17 healthy adults. Results showed that consuming cottage cheese with a meal led to a higher insulin response over four hours, and a higher insulin/glucose ratio over two hours, compared to the cod and soy proteins [52]. The cod protein meal resulted in a higher glucose response over 90 min, compared to cottage cheese and soy protein, but it evened out for all meals after two hours. These results suggest that in healthy subjects, dairy protein consumption at meals leads to more potent and quicker-acting glucoregulatory effects than lean fish protein or soy proteins. Interestingly, the early insulin peak at 40 min for all protein groups was similar, with insulin levels staying elevated for longer in the cottage cheese group. It is unclear if this effect was due to increased insulin production over time in response to the cottage cheese intake, or if it was from more efficient insulin clearance by the liver in the lean fish protein and soy protein isolate groups. A four-week study of 19 insulin-resistant adults compared a diet containing lean cod fish as the primary source of protein to a diet that consisted of a combination of animal proteins (dairy products, lean beef, veal, pork, and eggs). Results showed an improvement in insulin sensitivity and C-reactive protein in subjects consuming lean cod [53,54]. Taken together, these studies suggest that in healthy adults (and in those with insulin resistance), the effects of different protein sources are extremely divergent, with some being much better at potentiating the insulin response, and others having greater potential effects on glucose control, insulin sensitivity and/or insulin clearance.

Overall, the evidence from recent prospective cohort studies and RCTs has consistently shown that replacing animal proteins with plant proteins may be beneficial for glucose homeostasis and lowering T2DM risk. However, studies that ignore the vastly heterogeneous nature of large food groups and simply assess plant protein versus animal protein will undoubtedly miss critical caveats underlying the unique relationships between different protein sources and T2DM risk.

## 4. Different Effects of Milk, Meat, Fish, and Egg on Glucoregulatory Markers and Type 2 Diabetes Risk

Several prospective cohort studies on U.S. populations have found associations between animal protein intake and T2DM risk [55,56,57,58,59]. These associations are highly dependent on factors such as the source and type of animal protein, the type and amount of associated lipids consumed, and the amount and type of processing the animal product has undergone. A systematic review and meta-analysis of 20 studies (17 prospective cohorts and three case-control studies) investigated the associations between unprocessed red meat and processed meat consumption and the risk of incident T2DM. Results showed that intake of red meat was not associated with T2DM risk, while processed meat intake was associated with a 19% higher risk of T2DM [55]. The relationship between processed meat intake and T2DM risk is most likely due to non-protein compounds (e.g., sodium, nitrites, heme-iron, etc.), since the protein content and composition of processed and unprocessed meats are similar. The prospective research on other animal protein foods, such as eggs [60,61,62,63] and fish [64,65,66], has been equivocal and challenging to assess independently from their fat content. More qualitative analyses on the intake of different types of fish, particularly lean fish, fatty fish, and shellfish, have also found no consistent associations between intake and T2DM risk [67,68,69]. The prospective research studies on full-fat dairy products have been inconsistent, but the body of evidence regarding low-fat, fat-free, and fermented dairy products has consistently shown inverse associations with T2DM risk [49,70,71,72,73,74]. These associations have often been attributed to the direct effects of lowering fat intake on human health without much credence to the fact that lower fat content in a product can alter the bioavailability, activity and absorption kinetics of other nutrients, and bioactive compounds in that product.

Acute challenge studies regarding protein and fat intake show unique effects of animal protein types (e.g., cheese, egg, beef steak, etc.) on insulin secretion, which differ between healthy and T2DM populations. Food Insulin Index (FII) scores have been used to show considerable differences in insulin responses to different foods and meals. FII scores are a measure of the 2 h insulin response in healthy adults to a ~240 kcal portion of individual foods relative to a ~240 kcal portion of reference foods, such as white bread or glucose [29]. Therefore, if a researcher is using glucose as their reference food, a FII score of 50 means that the food tested will cause an insulin response that is one-half as large as an isocaloric amount of glucose. FII scores vary considerably for different protein sources (e.g., beef vs. tuna) and within protein sources that contain different macronutrient ratios (e.g., whole vs. skim milk) (Table 2) [29]. For example, a 240 kcal portion of cheddar cheese (15 g protein, 0.1 g carbohydrate, 20 g fat) resulted in a 36% higher 2 h insulin response than an isocaloric serving of egg (19.6 g protein, 0.5 g carbohydrate, 17.9 g fat), despite having similar macronutrient composition [75]. Cheddar cheese intake led to a similar insulin score as beefsteak (42 g protein, 0 g carbohydrate, 7 g fat), even though they contained very different levels of protein and fat. Overall, when matched for macronutrient and energy content, meat (beef and chicken) has been shown to be more insulinogenic than fish. When matched for energy content, higher protein and lower fat levels in milk, fish, and meat result in higher FII scores [29].

A study comparing high-FII or low-FII meals that had been matched for total energy, fats, protein, carbohydrates, and glycemic index showed that both sets of meals led to similar glycemic responses over the course of a single day [76]. However, the higher-FII meals led to a 53% insulin response over eight hours in healthy individuals, and a 41% higher insulin response in individuals with T2DM. These findings confirm that dietary choices can be an effective management tool for individuals with T2DM. The results also show that certain foods or food combinations that increase insulin secretion do not reduce blood glucose levels in a dose-dependent manner in healthy or T2DM subjects; this implies that other critical factors, such as the insulin sensitivity, insulin clearance rates, and non-insulin-dependent mechanisms of glucose uptake by muscle and adipose, must also be taken into account when assessing the impact of a food, meal or diet on glycemic response.

A study investigating the effects of adding 25 g of various animal proteins to a 50 g glucose meal in subjects with T2DM found that the addition of protein from lean beef, turkey, gelatin, egg white, cottage cheese, and fish all increased plasma insulin concentrations more than consuming glucose alone [11]. The addition of egg white increased the insulin response by nearly two-fold, while the addition of cottage cheese increased the insulin response by 3.6-fold. Despite the large increases in insulin secretion, only the additions of 25 g protein from turkey, gelatin, and cottage cheese were able to lower the postprandial glucose levels below that of ingesting glucose alone, while the addition of lean beef, egg whites, and fish did not lead to lower glucose levels than the control. In this study, and in similar research on healthy individuals, the intake of 25 g of egg white protein was shown to increase plasma glucose levels above baseline and resulted in the smallest insulin and glucagon responses among all of the proteins tested [11,77]. Cottage cheese intake led to the largest reduction in glucose levels, and the largest increase in insulin and glucagon response. Increased plasma glucagon levels are associated with the conversion of gluconeogenic precursors, such as gluconeogenic amino acids, into glucose. Dietary protein intake also causes an increase in glucagon secretion [78], and this effect has been shown to be exaggerated in subjects with T2DM [79]. However, the protein-dependent increase in glucagon does not correspond to an increase in blood glucose concentrations in healthy subjects or in subjects with T2DM, likely due to the proteins’ effects on insulin and incretin secretion and, in turn, insulin’s and GLP-1’s ability to suppress glucagon’s effects on gluconeogenesis and glycogenolysis [80,81,82].

Taken together, the findings on animal protein intake and T2DM risk convey the importance of not only assessing animal protein sources (e.g., beef, poultry, fish), but also protein types (e.g., milk, meat, egg), and the full composition of the foods and meals they are contained in. When animal protein sources have been compared in observational studies, acute challenge studies, and short-term intervention studies, the data consistently show that the higher intake of dairy foods (especially low-fat and fat-free dairy foods) improves glucose response and/or reduces T2DM risk to a greater extent than other commonly consumed animal protein sources.

## 5. Dairy Foods and Dairy Protein Supplements Are Associated with Improvements in Glucoregulatory Markers

Cow’s milk contains 3%–4% protein. Almost all of the proteins in milk and dairy products come from two major protein fractions, casein and whey protein, which make up approximately 80% and 20%, respectively, of proteins [83] (Table 1). Therefore, casein accounts for approximately 2.5%–3% of the total content of milk, while whey proteins accounts for around 1% of the total content of milk. The casein-to-whey ratio is similar in milk and yogurt, but varies greatly in commercial cheeses. Cottage cheese contains the highest ratio of casein to whey, followed by hard cheeses, and then soft cheeses. Dairy protein supplements, such as whey protein isolate, may range up to 95% protein, and may contain levels of bioactive proteins and peptides not normally seen in traditional dairy foods. Since milk, yogurts, cheeses, and dairy protein supplements all differ in their protein, carbohydrate, fatty acid, micronutrient, prebiotic, probiotic, and bioactive profiles, each dairy product will have different effects on glucoregulatory functions.

In healthy subjects, acute challenge studies show that milk intake leads to an approximately 3.5- to 4.4-times-higher insulin response than predicted by its glucose response [84]. Comparatively, in subjects with T2DM, milk intake has been shown to lead to roughly a five-fold greater increase in insulin response than expected, based on its glucose response [85]. These findings suggest that beta cells may retain their sensitivity or become more sensitized to specific proteins and nutrient combinations in individuals with compromised glucose sensing and signaling. When matched for 25 g carbohydrate content, the insulinogenic effects of milk in healthy adults were found to occur independent of the fat content, since both skimmed milk and whole milk led to similar glucose and insulin responses [84]. However, when a similar study in healthy subjects was conducted testing FII scores, which are matched for total energy content instead of carbohydrate content, the milkfat content did appear to make more than a two-fold difference in the insulinogenic effects of milk. This study tested isocaloric amounts of whole milk (3.5% milkfat), low-fat milk (1% milkfat), and fat-free milk and found that higher fat content in milk led to considerably lower postprandial insulin production (Table 2) [29].

Dairy foods have consistently shown promise for their effects on metabolism and glycemic control [86,87], but there are also many non-protein factors in dairy foods that can modulate their relationship with T2DM risk and management. Non-protein dairy components, such as magnesium, calcium, vitamin D in fortified dairy products, fatty acids, and probiotics in fermented dairy products, have been associated with lower T2DM risk [48,88,89], and it may be that particular combinations of these compounds have additive or synergistic effects on glucoregulatory outcomes. For example, a six-week study in subjects with T2DM found that consuming 300 g/day of a probiotic yogurt containing *Lactobacillus acidophilus* La5 and *Bifidobacterium lactis* Bb12 could improve fasting glucose levels, compared to baseline levels and compared to a conventional yogurt control [90]. Another study in subjects with T2DM showed that daily ingestion of 500 mL/day of vitamin D–fortified doogh led to improvements in glycemic status and fasting insulin levels after 12 weeks, compared to an unfortified control doogh [91,92]. Several other studies have shown that the ingestion of vitamin D alone can also improve glycemic control in subjects with T2DM by potentially affecting multiple aspects of both insulin secretion and sensitivity [93,94,95]. These studies may suggest that, in addition to the protein content and composition of dairy products, further glucoregulatory benefits can be achieved through the addition of probiotics and vitamin D to those products.

The concentrations of protein and protein components, such as EAAs, BCAAs, and potentially bioactive peptides, are much higher in dairy protein supplements than in dairy foods. In general, the higher proportion of BCAAs and the quicker rate of absorption of amino acids in protein supplements, compared to whole dairy foods, have been most commonly credited for their effects on metabolism and insulin secretion [81,96]. However, several studies have shown that whey protein’s effects on insulin and incretin secretion are modulated by its unique profile of bioactive peptides [97,98], which are released exogenously through food processing and endogenously through gastrointestinal digestion [98]. Whey proteins, caseins, and their bioactive peptides have all been shown to have potent glucoregulatory effects in healthy adults and in individuals with T2DM [97,99,100,101,102,103]. Mechanistically, bioactive peptides generated from the digestion of dairy proteins may have direct effects on insulin secretion, incretin secretion [96,104,105], and/or attenuation of incretin breakdown through inhibition of the incretin breakdown enzyme dipeptidyl peptidase 4 (DPP-4) [106,107].

The epidemiological evidence consistently shows that higher intake of dairy foods, such as milk, yogurt, and cheese, is associated with lower T2DM risk [70,71,72,73], but the epidemiological evidence investigating the intake of dairy protein supplements and T2DM risk is limited. Whey and casein protein supplements have traditionally been marketed for exercise- and recovery-related reasons, and not for glucose regulation or disease risk management. Therefore, the majority of studies investigating the effects of whey protein and casein protein on glucoregulatory function have been acute challenge studies and short-term intervention trials. We have previously reviewed the evidence from dairy food and dairy protein feeding studies in populations with T2DM, and found that both dairy foods and dairy protein supplements are associated with improvements in the glycemic status of subjects with T2DM [108]. Another recent systematic review of 10 weight-stable intervention studies, investigating the effects of higher dairy intake and insulin sensitivity in non-T2DM subjects, found that improvements in insulin sensitivity were only noticeable after 12 weeks of consuming a higher-dairy diet [109]. Of the 10 studies, nine looked at the effects of the higher intake of dairy foods, while only one looked at the effects of whey protein supplements on insulin sensitivity measures. Four of the studies, including the whey protein study [110], showed that higher dairy intake improved insulin sensitivity measures, while five studies showed a neutral effect. While dairy proteins are best known for their ability to potentiate insulin secretion, the data from this review suggests that long-term daily consumption of certain dairy foods may also have the potential to improve pathways relating to insulin sensitivity [109].

The insulinogenic effects of dairy products are extremely heterogeneous, with nearly a five-fold range in FII [29]. These effects are dependent on both their types and ratios of macronutrients, and on their bioactive constituents. Several factors other than protein content and quality mediate the relationship between dairy intake, glucose regulation and T2DM risk. Those factors are both insulin-dependent and insulin-independent, and neither are well understood. Therefore, more RCTs, along with the use of valid biomarkers, are needed to better understand the insulin-potentiating effects of different dairy products, as well as to determine the mechanisms responsible for the insulin-sensitizing and insulin-independent effects of dairy foods and dairy protein supplements on glucoregulatory markers.

## 6. Gaps in Existing Knowledge and Future Research Directions

Major knowledge gaps exist between the source of dietary protein and the management of T2DM. Several large prospective studies have shown an inverse relationship between plant protein and dairy protein intake and T2DM risk, yet the nutrient and bioactive composition of these foods groups are very different. Additionally, the nutrient and bioactive composition of individual foods in each of these groups are extremely heterogeneous, with some containing mostly fat and others containing mostly protein or carbohydrates. Acute challenge studies show greater effects for dairy protein foods than plant protein foods on insulin and incretin secretion, but the differences between protein sources on other aspects of glucose regulation, such as insulin sensitivity and hepatic glucose clearance, are not well understood. Longer-term RCTs that manipulate protein intake variables are necessary to determine which glucoregulatory effects persist over time, and which populations (e.g., healthy, overweight, insulin-resistant, T2DM) would benefit most from increasing dairy protein and/or plant protein food intake. Furthermore, T2DM has multiple pathophysiologies and treatment plans; accordingly, different individuals will have different responses to different foods and dietary manipulations. Moving from a paradigm of population-based nutrition research to one of more personalized nutrition, based on an individual’s particular nutrigenetic and nutrigenomic profile, would greatly enhance the ability of clinicians to optimize protein intake. In order to recommend the ideal source, type, and amount of dietary protein for managing blood glucose levels, there are several key questions that need to be answered to best optimize protein intake in order to close the gap in the existing knowledge.

How does an individual’s phenotypic status, such as age, weight, gender, activity level, disease status, etc., affect their glucoregulatory responses to protein foods and protein supplements?

How does an individual’s nutrigenetic status and nutrigenomic status affect their glucoregulatory status?

How does the timing of ingestion, such as pre-meal, with meal, and/or between meals, affect a protein source’s glucoregulatory abilities in different populations?

How does the frequency of ingestion, such as a single daily dose, before every meal, and/or before carbohydrate-rich meals only, affect a protein source’s glucoregulatory abilities in different populations?

How do different combinations and concentrations of non-protein components, such as fat, carbohydrates, micronutrients, and bioactives, in protein foods affect their glucoregulatory abilities in different populations?

What are the major glucoregulatory effects of fermentation, and what are the differences between various probiotic strains and combinations of strains on the glucoregulatory properties of protein foods? 

How do different ingredients and food processing techniques affect a protein food’s glucoregulatory abilities in different populations?

What are the long-term glucoregulatory effects of ingesting different types and doses of protein supplements in different populations?

What are the effects of protein foods and protein supplements in combination with and compared to common glucoregulatory medications, such as insulin secretagogues, insulin sensitizers, incretin analogs and agonists, etc.?

## 7. Conclusions

The protein quantity and quality of foods are contributing factors to their effects on glucose control, but foods are much more complex than a single nutrient, or even the sum of their individual nutrients. Many dietary factors, nutritive and/or bioactive, mediate the relationship between food intake and health. For example, plant protein foods contain fibers and numerous phytochemicals that have been associated with an array of beneficial health outcomes, while processed meats containing sodium, nitrites, and heme-iron have been linked with less-favorable health outcomes. Dairy foods, such as milk, yogurt, and cheese, contain a food matrix rich in high quality proteins, calcium, magnesium, potassium, trans-palmitoleic fatty acids, low-glycemic-index sugars, and oligosaccharides which have all been shown to have beneficial effects on aspects of glucose control, insulin secretion, insulin sensitivity, and/or T2DM risk [86,98,111,112]. The dairy protein supplements whey and casein do not contain as many non-protein compounds as dairy foods, but do contain higher concentrations of insulinogenic amino acids, BCAAs, and bioactive peptides, which have consistently been associated with beneficial glucoregulatory outcomes in healthy and T2DM populations [97,98,113,114]. Dietary recommendations for dairy intake that focus only on amount (e.g., two to three servings of dairy per day) may be adequate for general health, but are much too ambiguous for those attempting to improve their blood glucose control. Further focus on product type (e.g., milk, cheese, yogurt, whey protein); product characteristics (e.g., full-fat, low-fat, fat-free, fortified, fermented, etc.); and protein properties (e.g., BCAA content, EAA content, bioactive proteins and peptides) is necessary to better determine a product’s glucoregulatory abilities. Lastly, in order to best optimize protein intake for glucose regulation, the amount, source, and type of food product or supplement should be personalized to match to the individual’s lifestyle, medications, glucoregulatory abilities, and disease status.

## Figures and Tables

**Table 1:** Compositional differences between commonly consumed animal proteins.

Protein Source	Cow’s Milk	Meat (Beef, Poultry, Pork)	Fish (Cod, Salmon, Trout)	Eggs (Chicken)
Major Protein Groups and Types	Caseins (80%)	Myofibrillar (55%–60%)	Myofibrillar (70%–80%)	Egg White (50%–60%)
	alpha-caseins beta-caseins kappa-caseins gamma-caseins	myosin actin titin nebulin tropomyosin troponins	myosin actin titin nebulin tropomyosin troponins	ovalbumin ovotransferrin ovomucoid ovomucin lysozyme
	Whey Proteins (20%)	Sarcoplasmic (20%–30%)	Sarcoplasmic (25%–30%)	Egg Yolk (40%–50%)
	beta-lactoglobulin alpha-lactalbumin serum albumin immunoglobulins lactoferrin transferrin	globins cytochromes metabolic enzymes	globins cytochromes metabolic enzymes	livetins lipovitellins lipoproteins phosvitin
		Stromal (10%–20%)	Stromal (5%–10%)	
		collagen elastin	collagen elastin	
Branched-Chain Amino Acids (BCAAs) and Other Essential Amino Acids (EAAs)—value per gram of protein	BCAAs (mg)	BCAAs (mg)	BCAAs (mg)	BCAAs (mg)
	Ile 40–57 Leu 75–107 Val 53–73	Ile 32–55 Leu 56–93 Val 36–59	Ile 46–53 Leu 80–94 Val 51–59	Ile 51–56 Leu 84–91 Val 66–72
	Other EAAs	Other EAAs	Other EAAs	Other EAAs
	His 25–37 Lys 65–93 Met 20–30 Phe 40–60 Thr 32–47 Trp 10–17	His 24–42 Lys 60–108 Met 19–30 Phe 30–46 Thr 28–51 Trp 04–14	His 28–34 Lys 91–106 Met 29–34 Phe 39–45 Thr 43–51 Trp 11–13	His 24–26 Lys 70–76 Met 29–32 Phe 52–57 Thr 43–47 Trp 13–14

Data sources: Protein Groups and Types—[19,20,21,22]; Essential Amino Acid Content—USDA Agricultural Research Service, National Nutrient Database for Standard Reference Release 28 [23]. Full Reports for: 01077, Milk, whole, 3.25% milkfat, with added vitamin D; 01151, Milk, nonfat, fluid, without added vitamin A and vitamin D (fat-free or skim); 13974, Beef, chuck eye roast, boneless, America’s Beef Roast, separable lean only, trimmed to 0% fat, select, raw; 13498, Beef, ground, 70% lean meat/30% fat, raw; 05062, Chicken, broiler or fryers, breast, skinless, boneless, meat only, raw; 10219, Pork, fresh, ground, raw; 15015, Fish, cod, Atlantic, raw; 15076, Fish, salmon, Atlantic, wild, raw; 15114, Fish, trout, mixed species, raw; 01123, Egg, whole, raw, fresh.

**Table 2:** Functional and qualitative differences between commonly consumed animal proteins.

Protein Source	Cow’s Milk	Meat (Beef, Poultry, Pork)	Fish (Cod, Salmon, Trout)	Eggs (Chicken)
Protein Functions	Nutrient transfer from mother to offspring	Structural	Structural	Structural
	Immune and non-immune protection	Locomotion	Locomotion	Nutrient transfer from mother to offspring
	Signaling for offspring growth and development	Muscle metabolism	Muscle metabolism	Signaling for embryonic development
Protein Quality Scores:				
Protein Efficiency Ratio (PER)	2.5	2.7–2.9	2.7	3.8
Biological Value (BV)	91	80	83	100
Protein Digestibility Corrected Amino Acid Score (PDCAAS)	1.00	0.92	0.98	1.00
Digestible Indispensable Amino Acid Score (DIASS)	1.3	1.1–1.3	None-Given	1.3
Food Insulin Index—240 kcal portion (% relative to 240 kcal of glucose)	Whole Milk (24%) Low-fat Milk (34%) Fat-Free Milk (60%)	Chicken, no skin (17%) Chicken, w/skin (19%) Beef steak (37%)	Tuna in oil (16%) Tuna in water (26%) White fish filet (43%)	Poached egg (23%)

Data sources: Protein Quality Scores—[24,25,26,27,28] Food Insulin Index [29].

## Abbreviations

T2DM	Type 2 Diabetes Mellitus
BCAA	Branched-Chain Amino Acid
EAA	Essential Amino Acid
AMDR	Acceptable Macronutrient Distribution Ranges
RCT	Randomized Controlled Trial
DGAC	Dietary Guidelines Advisory Committee
DIAAS	Digestible Indispensable Amino Acid Score
PDCAAS	Protein Digestibility-Corrected Amino Acid Scores
PER	Protein Efficiency Ratio
BV	Biological Value
FII	Food Insulin Index
GIP	Glucose-Dependent Insulinotropic Peptide
GLP-1	Glucagon-Like Peptide-1
Dipeptidyl Peptidase 4	DPP-4
